# Use of Targeted Exome Sequencing in Genetic Diagnosis of Chinese Familial Hypercholesterolemia

**DOI:** 10.1371/journal.pone.0094697

**Published:** 2014-04-10

**Authors:** Wen-Feng Wu, Li-Yuan Sun, Xiao-Dong Pan, Shi-Wei Yang, Lv-Ya Wang

**Affiliations:** 1 Beijing Anzhen Hospital Affiliated with Capital Medical University, Beijing, China; 2 Department of Atherosclerosis, Beijing Institute of Heart Lung and Blood Vessel Diseases, Beijing, China; Institut Jacques Monod, France

## Abstract

Familial hypercholesterolemia is an autosomal dominant inherited disease characterized by elevated plasma low-density lipoprotein cholesterol (LDL-C). It is mainly caused by mutations of the low-density lipoprotein receptor (*LDLR*) gene. Currently, the methods of whole genome sequencing or whole exome sequencing for screening mutations in familial hypercholesterolemia are not applicable in China due to high cost. We performed targeted exome sequencing of 167 genes implicated in the homozygous phenotype of a proband pedigree to identify candidate mutations, validated them in the family of the proband, studied the functions of the mutant protein, and followed up serum lipid levels after treatment. We discovered that exon 9 c.1268 T>C and exon 8 c.1129 T>G compound heterozygous mutations in the *LDLR* gene in the proband derived from the mother and father, respectively, in which the mutation of c.1129 T>G has not been reported previously. The mutant LDL-R protein had 57% and 52% binding and internalization functions, respectively, compared with that of the wild type. After 6 months of therapy, the LDL-C level of the proband decreased by more than 50% and the LDL-C of the other family members with heterozygous mutation also reduced to normal. Targeted exome sequencing is an effective method for screening mutation genes in familial hypercholesterolemia. The exon 8 and 9 mutations of the *LDLR* gene were pedigree mutations. The functions of the mutant LDL-R protein were decreased significantly compared with that of the wild type. Simvastatin plus ezetimibe was proven safe and effective in this preschool-age child.

## Introduction

Familial hypercholesterolemia (FH; MIM #143890) is a serious single-gene, dominant genetic disease. The main clinical manifestations are significantly elevated plasma low-density lipoprotein cholesterol (LDL-C) levels. The pathological basis of FH is LDL receptor (*LDLR*; MIM #606945) gene mutations that cause cholesterol metabolism dysfunction. Clinically, FH is divided into homozygous and heterozygous phenotypes. The latter is relatively common and leads to premature coronary heart disease; it occurs in 1∶500 people in most countries. The former is rare, occurring in 1∶1,000,000 births; however, its symptoms are severe [1], [2]. However, as the most populous country in the world, China may bear a heavy burden of this genetic disorder. However, there is no applicable genetic screening method in clinical practice in China.

To date, 3 genes *LDLR*, apolipoprotein B (APOB), and proprotein convertase subtilisin/kexin 9 (PCSK9) have been accepted [3]. Reported mutations of FH widely spread over the coding regions of these causative genes. Genetic screening through traditional approaches, such as direct sequencing is therefore difficult. A high-throughput and cost-effective method to detect the genetic defects is needed. Whole exome sequencing has been proved to be a powerful tool to discover novel disease-related genes or genetic mutations in large genomic regions [4]–[6]. With the progresses on next-generation sequencing (NGS) and bioinformatics, it has been demonstrated to have higher efficiency but lower cost comparing with whole gene sequencing. However, the astronomical information amount, subsequent arduous data processing and large cost limit its widely application in practice in China. In this study, we utilized targeted exome sequencing (TES) to study genetic defects in one family, in order to establish a strategy feasible to genetic diagnosis of FH patients. We also studied the binding and internalization functions of the mutant protein and followed up the effect of lipid-lowering therapy in the pedigree.

## Materials and Methods

### Ethics Statement

The study was reviewed and approved by the Ethical Committee of Beijing Anzhen Hospital, and all participants signed an informed consent form. For those aged <18 years, written consent was obtained from either parent.

### Study Population and Data Collection

**Figure 1:**
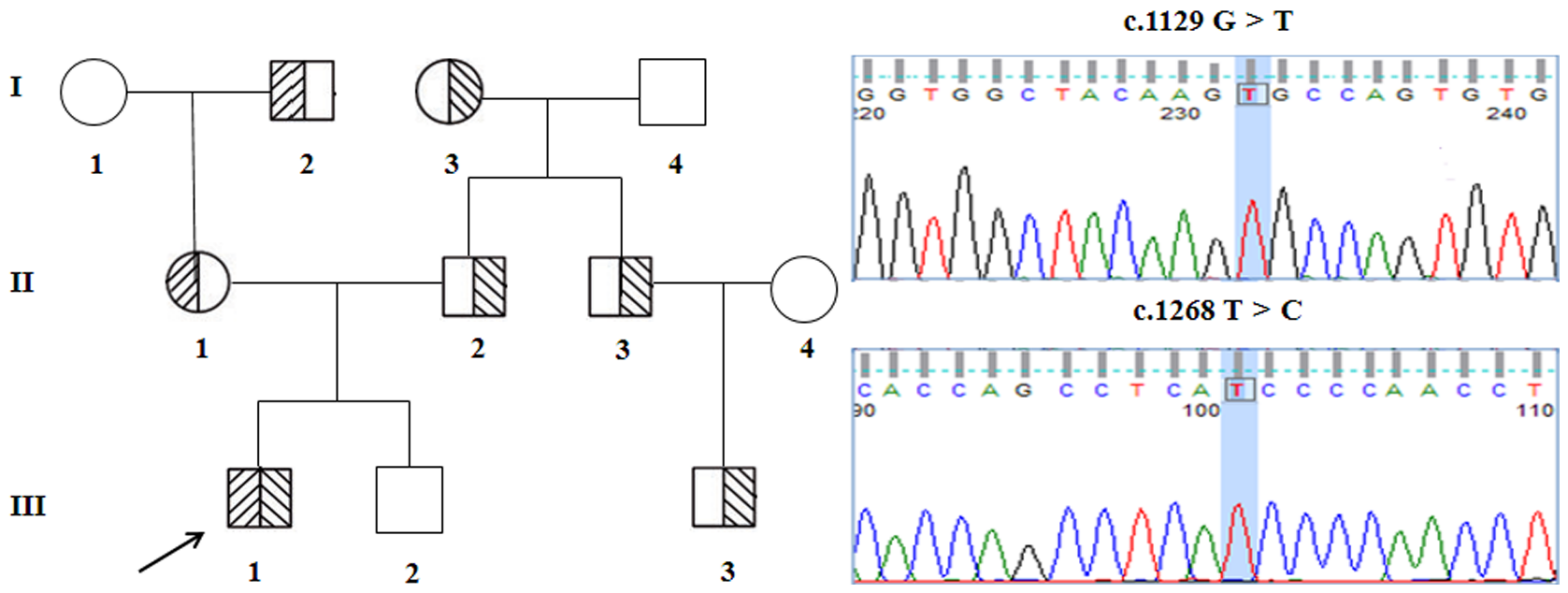
Pedigree and mutations of a hypercholesterolemic family. Arrow denotes the proband. Shaded and partially shaded shapes denote compound heterozygotes and heterozygotes with different mutations, respectively. The exon 8 c.1129 G>T and exon 9 c.1268 T>C mutations are depicted on the right.

**Figure 2:**
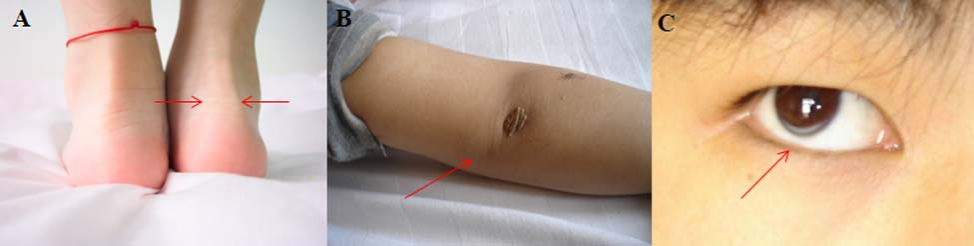
Clinical manifestations of the proband. (A) Achilles tendon thickening. (B) Cutaneous xanthomas on the elbow region. (C) Arcus corneae.

This study was part of a large study aimed at screening FH mutations using TES in the Han Chinese population. An FH family was recruited at Beijing Anzhen Hospital in February 2013 (Figure 1). The proband (III-1) was a 5-year-old boy with distinct clinical features of skin xanthoma near the elbow, lap, and hips (Figure 2); his parents had no apparent clinical features but had high TC and LDL-C upon physical examination. Homozygous FH was diagnosed based on the following criteria: (1) plasma or serum LDL-C>10 mmol/L; (2) presence of tendon and cutaneous xanthomas at an early age; (3) autosomal inheritance of hypercholesterolemia in relatives; (4) presence of primary hypercholesterolemia in the parents of the proband [7]. Secondary causes of hypercholesterolemia were excluded. The clinical features of members in the FH family were investigated (Table 1).

**Table 1:** Clinical Data of the Family.

ID	Sex	Age (years)	TC (mmol/L)	TG (mmol/L)	LDL-C (mmol/L)	HDL-C (mmol/L)	Arcus corneae	Cutaneous xanthoma	Tendon xanthoma	CHD
grandmother	F	57	4.64	1.65	3.24	1.16	No	No	No	No
grandfather	M	57	6.70	0.79	5.00	1.34	Yes	No	No	No
grandmother	F	66	7.89	0.98	5.54	1.12	Yes	No	No	No
grandfather	M	68	5.12	1.09	3.11	1.92	No	No	No	No
mother	F	28	8.15	1.45	6.26	1.23	Yes	No	No	No
father	M	34	7.56	1.50	5.33	1.55	Yes	No	No	No
uncle	M	44	8.29	1.62	6.53	1.34	Yes	Yes	No	No
aunt	F	42	4.45	1.32	3.12	1.74	No	No	No	No
proband	M	2	24.65	1.35	14.80	2.86	Yes	Yes	No	No
sibling	M	0	4.51	0.51	2.60	1.54	No	No	No	No
counsin	M	23	6.56	1.14	4.60	1.48	No	No	No	No
Normal range			≤5.20	≤1.70	≤4.12	≥1.04				

Abbreviations: TC, total cholesterol; HDL-C, high-density lipoprotein cholesterol; LDL-C, low-density lipoprotein cholesterol; TG, triglyceride; M, male; F, female; CHD, Coronary heart disease.

### Blood Sampling and Measurements

Blood samples were obtained from participants after a 12-h fast. TC, triglycerides (TG), LDL-C, and high-density lipoprotein cholesterol (HDL-C) were measured using routine commercial kits (Beckman Coulter, Brea, CA) and a Beckman AU 4500 automated biochemical analyzer (Beckman Coulter).

### Illumina Library Construction

Proband genomic DNA was extracted from whole blood using a DNA Extraction Kit (TianGen, Beijing, China) according to the manufacturer's instructions. DNA quantification was performed using a NanoDrop 2000 unit (Thermo Fisher Scientific, Wilmington, DE). DNA (minimum 3 μg) was used for the indexed Illumina libraries as per the manufacturer's protocol. A final library size of 350–400 bp, including adapter sequences, was selected.

### Enrichment and Sequencing of Disease Genes

In total, 167 disease genes, including the genes relevant with blood lipids [8] and cholic acid metabolism (Table S1) were selected by a gene capture strategy using a GenCap Custom Enrichment Kit (MyGenostics, Beijing, China) according to previously described technologies [9], [10]. The capture experiment was conducted according to the manufacturer's protocol. Briefly, 1 μg DNA library was mixed with Buffer BL and a GenCap hypercholesterolemia probe (MyGenostics) and heated in a polymerase chain reaction (PCR) machine at 95°C for 7 min and 65°C for 2 min. 23 μL of the 65°C pre-warmed Buffer HY (MyGenostics) was added; the mixture was held at 65°C with the PCR lid heat on for 22 h for hybridization. 50 μL MyOne beads (Life Technology, Carlsbad, CA) were washed in 500 μL 1× binding buffer thrice and re-suspended in 80 μL 1× binding buffer. 64 μL 2× binding buffer was added, the mixture transferred into a tube containing 80 μL MyOne beads, and spun for 1 h on a rotator. We washed the beads once with WB1 buffer at room temperature for 15 min and WB3 buffer thrice at 65°C for 15 min. Elution buffer was used to elute the bound DNA, which was amplified as follows: 98°C for 30 s; 98°C for 25 s, 65°C for 30 s, 72°C for 30 s (15 cycles); 72°C for 5 min. We purified the PCR product using SPRI beads (Beckman Coulter) using the manufacturer's protocol. Enrichment libraries were sequenced on an Illumina HiSeq 2000 sequencer (Illumina, San Diego, CA) for 100-bp paired reads.

### Bioinformatics Analysis

After sequencing, we retrieved high-quality reads from raw reads by filtering out low-quality reads and adaptor sequences using the Solexa QA package [11] and cutadapt program (http://code.google.com/p/cutadapt/), respectively. We used the SOAPaligner program [12] to align the clean read sequences to the human reference genome (hg19).

To detect exon duplication and deletions, the coverage of each position was plotted by the base positions. Higher coverage of a region denoted duplication; uncovered regions indicated deletions. After removing duplicates with Picard software [13], single-nucleotide polymorphisms (SNPs) were identified using the SOAPsnp program [12] (http://soap.genomics.org.cn/soapsnp.html). Subsequently, reads were realigned to the reference genome using the Burrows–Wheeler alignment program [14], and insertions or deletions (InDels) were identified with the Genome Analysis Toolkit [15] (http://www.broadinstitute.org/gsa/wiki/index.php/Home_Page). We annotated identified SNPs and InDels using the Exome-assistant program (http://122.228.158.106/exomeassistant). Short read alignment and candidate SNP and InDel validation was performed using MagicViewer [16]. We used the PolyPhen, SIFT, PANTHER and PMUT algorithms to evaluate non-synonymous variants to determine pathogenicity [17]. Sequencing data were deposited in NIH Short Read Archive (SRR1169449).

### Expanded Validation

DNA samples from all family members were obtained for Sanger sequencing. We also PCR-amplified the coding regions of the mutations identified as described for conventional direct sequencing. We used an ABI 3500 Genetic Analyzer (Applied Biosystems, Foster City, CA) to cycle-sequence purified PCR products. We analyzed the Sanger sequencing results using Mutation Surveyor (Softgenetics, State College, PA), reconfirming them using the same procedure.

### Construction of Mutant

The C356G LDL-R mutant was constructed according to the protocol previously described [18].

### Functional Studies of the Mutant

LDL-R binding and internalization functions were measured as previously describe [18].

### Lipid-lowering Therapy, Blood Lipid Target, and Follow-up

**Figure 3:**
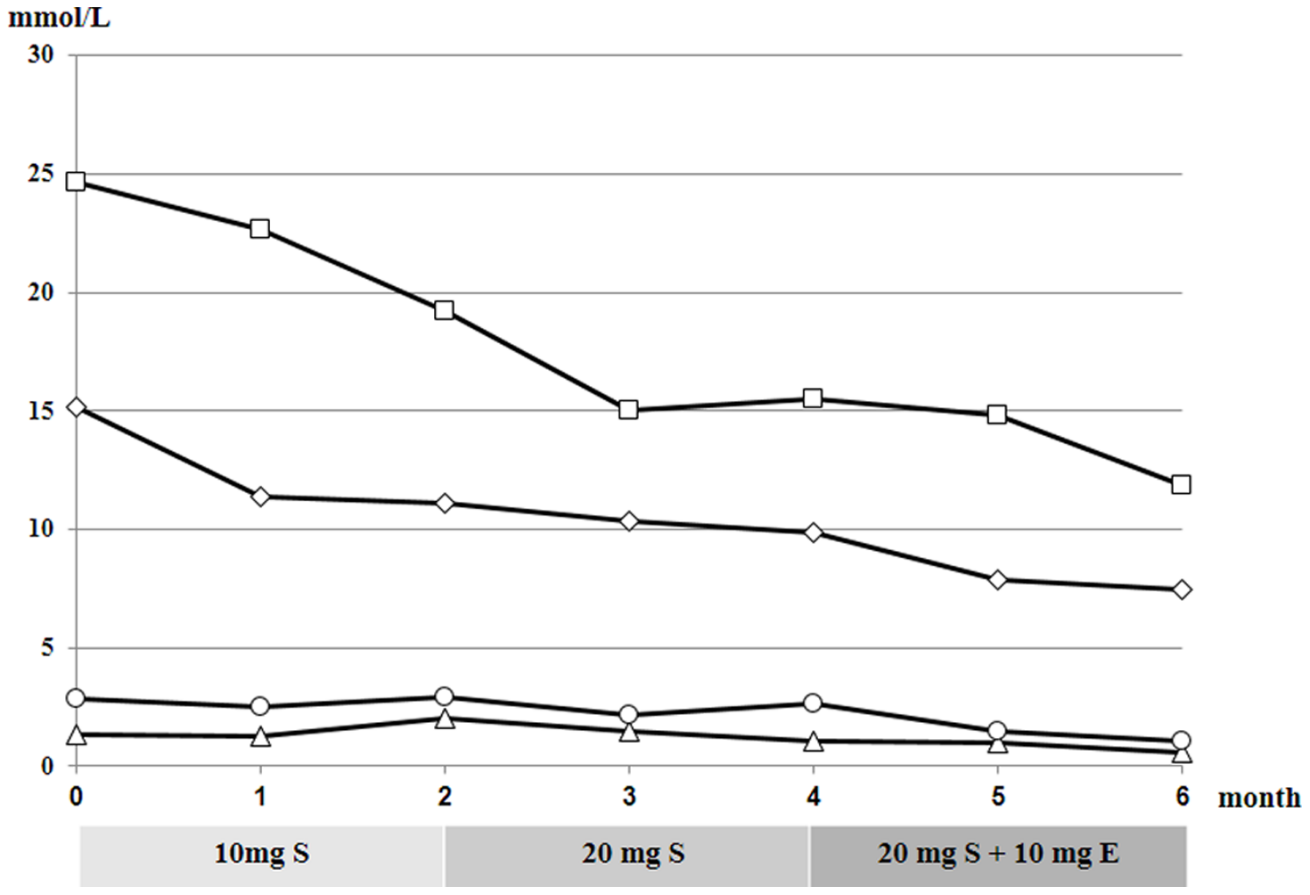
Serum lipid changes after lipid-lowering therapy. Plasma concentrations of TC (square), TG (triangle), LDL-C (diamond), and HDL-C (circle) in the proband. S, simvastatin; E, ezetimibe.

According to whether the proband reached the target ≥50% LDL-C reduction from baseline [19], we treated him with 10 and 20 mg simvastatin daily for 2 months, and then 20 mg simvastatin plus 10 mg ezetimibe daily for the next 2 months (Figure 3). Heterozygous patients in the family received 20 mg simvastatin daily.

Blood lipids were re-examined every month until 6 months later. The subjects were followed for serious adverse reactions.

## Results

### General Clinical Data

The general clinical data of the family are listed in Table 1. TC and LDL-C levels in the proband (III-1) were extremely high before treatment and he exhibited typical clinical features. The maternal grandfather (I-2), paternal grandmother (I-3), mother (II-1), father (II-2), uncle (II-3), and cousin (III-3) had a clinical diagnosis of heterozygous FH and high TC and LDL-C levels when recruited; only II-3 had cutaneous xanthoma. Other family members had normal serum lipid levels. All subjects had normal HDL-C levels, no tendon xanthoma, and we confirmed that there was no evidence of coronary heart disease.

### TES Identification of Candidate Mutations

**Figure 4:**
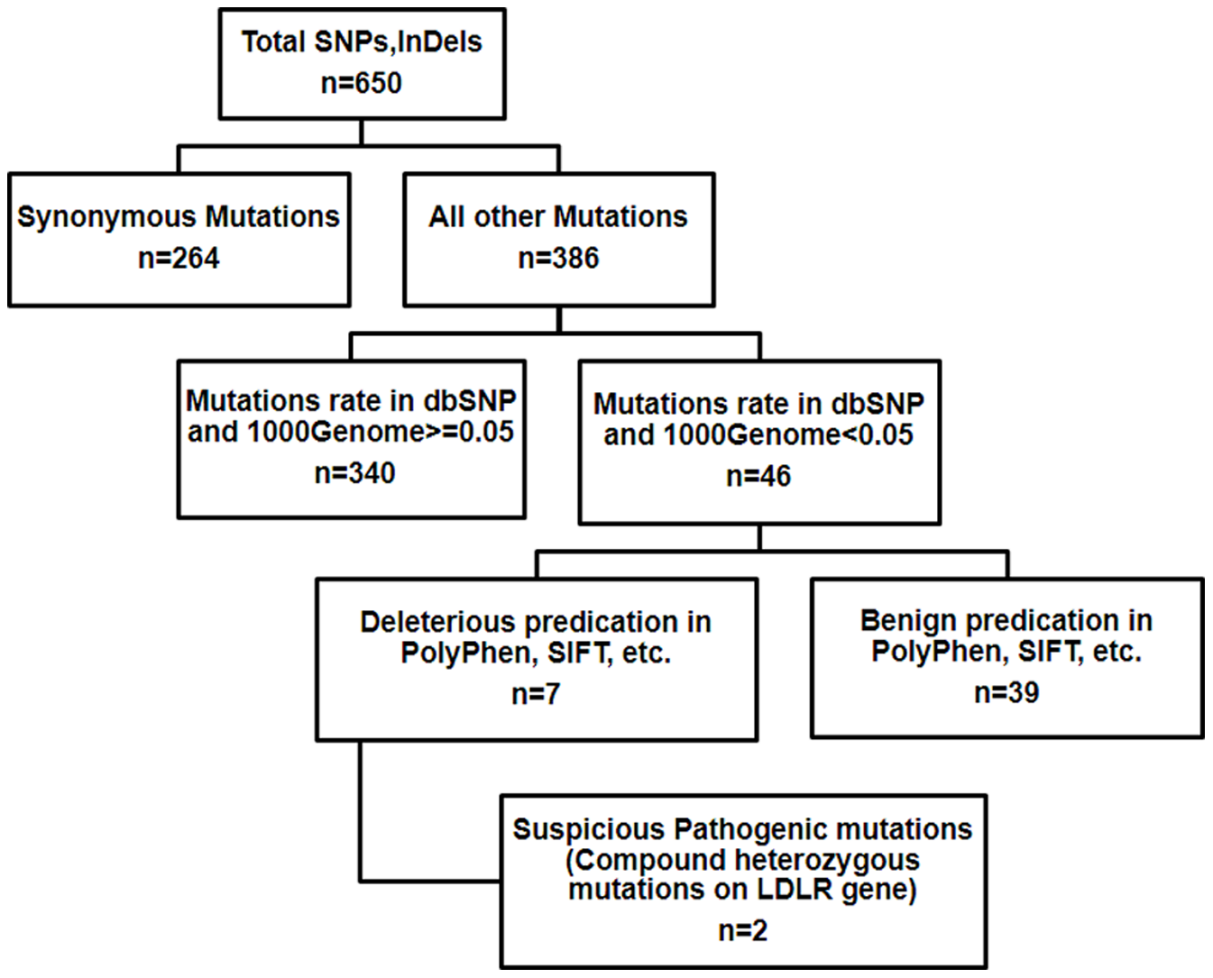
Flow chart of the data analysis.

We performed TES of 167 genes implicated in FH. The average sequencing depths on the targeted regions of samples that underwent TES was 640.32 (Table S2). The sample had 96.9% coverage of the targeted regions. Meanwhile, there was 92.3% and 89.3% coverage of targeted exons for 10 and 20 reads, respectively. Via SOAPsnp [13], an average 650 variants were identified in the sample. Among them were 386 non-synonymous variants, missense, nonsense, and splicing variants. This was narrowed down to 46 by excluding variants reported in HapMap 28 and the SNP release of the 1000 Genome Project with minor allele frequency>0.05. For missense variants, computational prediction by PolyPhen, SIFT, PANTHER, PMUT and consistency of genetic transmission mode further narrowed the number of candidate mutations to <8. For the 7 coding InDels initially identified in the sample using the Genome Analysis Toolkit program [15], we identified 2 compound heterozygous variants(I402T and C356G) on LDLR gene that correlated with the disease phenotype (Figure 4).

### Expanded Familial Validation and Sanger Sequencing Confirmation

We validated TES results from the proband to the entire family using Sanger sequencing. Sanger sequencing of the coding region confirmed that the I402T and C356G mutations were transmitted from the paternal grandmother (I-3) and the maternal grandfather (I-2), respectively. And, the heterozygous *LDLR* mutation I402T was carried by the father (II-2), uncle (II-3), and cousin (III-3), while the heterozygous *LDLR* mutation C356G was carried by the mother (II-1). But the sibling (III-2) carried no *LDLR* mutation. So the proband (III-1) carried 2 compound heterozygous *LDLR* mutations (C356G and I402T) (Figure 1). Furthermore, we performed co-segregation analysis to confirm the extracted mutations in the pedigree. All mutations were confirmed to co-segregate well with the disease in the family.

### Effect of C356G *LDLR* Mutation on LDL-R Function

**Figure 5:**
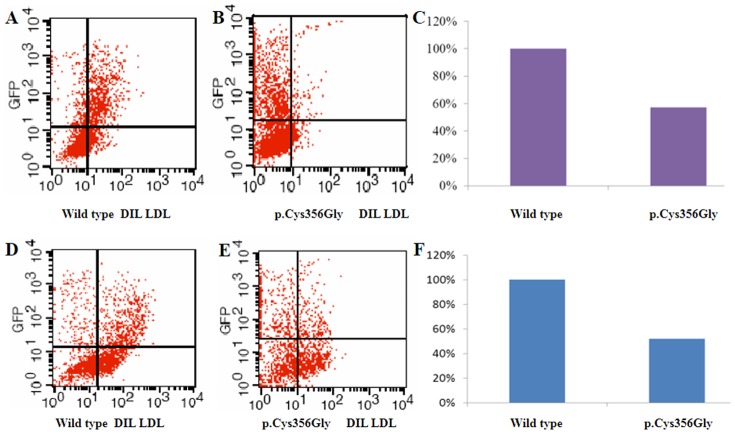
Characterization of C356G mutation LDL-R binding and internalization function. (A, D) Wild-type LDL-R binding and internalization function. (B, E) C356G LDL-R binding and internalization function. (C, F) Histogram of wild-type and C356G mutant LDL-R binding and internalization function.

The effects of the C356G mutation on LDL-R function was investigated by transient expression in HEK-293 cells. To measure LDL-R binding and internalization functions, HEK-293 cells were harvested 48 h after transfection and analyzed by flow cytometry. Analyses of antibody-labeled cells and measurement of activity revealed that receptors with the C356G mutation had 57% binding and 52% internalization activity compared with that of the wild-type receptor. The majority of EGFP-positive cells could not bind and internalize LDL effectively (Figure 5, Table 2).

**Table 2:** LDL-R Function Comparisons.

Group	LDL-R function analysis (geometric mean fluorescence intensity)	
	LDL-R binding ability (%)	LDL-R internalization ability (%)
Wild-type^a^	100	100
p.Cys356Gly mutant	57±9	52±10

a RefSeq: NP_000518.1., NM_000527.4.

Abbreviations: LDL-R, Low-density lipoprotein receptor.

### Family Lipid Level Follow-up

Lipid-lowering therapy was started upon the proband being diagnosed with FH. He was initially given 10 mg simvastatin daily to treat hyperlipidemia, but after 2 months, his LDL-C decreased by only 25% from baseline. We increased the dose to 20 mg daily; 2 months later, his LDL-C levels had decreased by about 35% from baseline, meaning the target level still had not been achieved. Therefore, we combined 20 mg simvastatin with 10 mg ezetimibe daily. At the end of the fifth month, his LDL-C levels had declined by 50% as compared to baseline and remained at about 7.4 mmol/L (Figure 3). After 6 months, lipid levels in heterozygous patients in the family had decreased to normal levels (Table 3). All subjects experienced no adverse reaction during treatment.

**Table 3:** Lipid Levels of Heterozygous Family Members after 6-month 20 mg Simvastatin Treatment.

Case No	Age (years)	Sex	TC (mmol/L)	TG (mmol/L)	HDL-C (mmol/L)	LDL-C (mmol/L)	LDL-R mutation
I-2	57	M	5.20	0.93	1.09	3.10	p.Cys356Gly (c.1129T>G)
I-3	66	F	4.75	1.21	1.13	3.87	p.IIe402Thr (c.1268T>C)
II-1	28	F	4.47	1.47	1.26	4.02	p.Cys356Gly (c.1129T>G)
II-2	34	M	5.10	0.96	1.53	3.41	p.IIe402Thr (c.1268T>C)
II-3	44	M	5.01	1.38	2.01	4.11	p.IIe402Thr (c.1268T>C)
III-3	23	M	3.87	1.19	1.67	3.17	p.IIe402Thr (c.1268T>C)
Normal range			≤5.20	≤1.70	≥1.04	≤4.12	

Abbreviations: TC, total cholesterol; HDL-C, high-density lipoprotein cholesterol; LDL-C, low-density lipoprotein cholesterol; LDL-R, LDL receptor; TG, triglyceride; M, male; F, female.

## Discussion

Estimated with the prevalence of heterozygous 1/500, there should be about 2.6 million potential FH patients in China, but the definitions of patients and potential patients are inconsistent. The first reason is that both doctors and patients have poor understanding of FH, and serum lipid levels are not examined during routine physical examination of children. On the other hand, only a few laboratories in China have the capability for detecting gene(s) mutations of FH. Therefore, a significant number of FH patients are not diagnosed and treated in a timely manner. Foreign research has shown that early diagnosis and intervention can delay the occurrence of complications of atherosclerosis and coronary heart disease in FH patients [20]–[22]. Traditional screening for FH mutations is also infeasible because distribution of the mutation sites is scattered. Whole exome sequencing has been proved to be a powerful tool to discover novel disease-related genes or genetic mutations in large genomic regions [4]–[6], but clinical application in China is rarely accepted due to high cost. In the present study, we carried out TES for 167 genes implicated in FH and found that the average sequencing depth of the target genes was >640, coverage of the target region was about 97%. The data from sequencing was about 650 Mb one sample (Table S2), equal to about 10% of whole exome sequencing. Therefore, we have proved that deep exome sequencing of target 167 genes is a fast and efficient method to assist the diagnosis. The cost was saved at least 50% compared with whole exome sequencing, even less the amount of work saved. Notably, only a single sample from the proband was sufficient for identifying the causative mutation in the family; finalization could be enhanced using intra-familial mutation validation and co-segregation analysis. Therefore, there are 3 advantages of TES: 1) the genetic screening range can be reduced greatly; 2) data analysis can be performed with high efficiency; 3) it is much less expensive than whole exome sequencing.

The latest version of the LDL-R FH database of the University College London (www.ucl.ac.uk/ldlr, accessed Jan 1, 2014) states that >1125 *LDLR* gene variants worldwide, including different point mutations, small and large fragment deletions, insertions, rearrangements, and many new mutations, were recently discovered. The I402T mutation, transmitted from the paternal grandmother in the present study, has been reported in Switzerland, France, and Malaysia [23]–[25], with binding and internalization functions of the mutant LDL-R protein decreasing to 59% and 54%, respectively, compared with the normal protein [26]. The C356Y mutation (nucleotide change to c.1130G>A) was first reported by Ekstrom in 1998 [27], but the C356G mutation (nucleotide change to c.1129T>G) has not been reported previously. In this study, we concentrated on the effect of the novel C356G mutation on LDL-R function. Flow cytometry revealed that receptors with the C356G mutation had 57% binding activity compared with that of the wild-type receptor. The mutant receptor also displayed 52% internalization function compared to that of the wild-type. In the LDL-R protein, the C356G mutation is located in the epidermal growth factor precursor homology domain B region, a disulfide- and cysteine-rich region. As this sequence is highly conserved, it may play an important functional role. It has been proven that the cysteine-to-glycine mutation has a marked effect on protein folding, impairing receptor function [26]. The epidermal growth factor precursor homology domain has been implicated in low-pH receptor recycling and lipoprotein release [28]; therefore, mutations in this region will disrupt the natural primary function of the uptake of cholesterol-carrying particles into cells, thus lipids remain in the blood, resulting in hyperlipidemia.

The proband was diagnosed with compound heterozygous FH and was administrated with cholesterol synthesis inhibitor, simvastatin (dose titrated from 10 to 20 mg daily), accompany with diet therapy, though evidence of simvastatin use in child is limited. Four months later, the LDL-C decreased just by less than 50%, did not reach the target. We assumed that such a reduction amount may arise from either possible statin escape [29] or the sole blocking function of cholesterol synthesis but not cholesterol absorption. We combined simvastatin with the cholesterol absorption inhibitor ezetimibe (10 mg daily); after 1 month, his LDL-C levels were reduced by 50% from baseline, consistent with the report by Catapano et al [30]. The therapy failed to lower LDL-C down to normal range. Nevertheless, the target (reduction ≥ 50%) recommended by the guideline was eventually met [19]. During treatment, the skin xanthoma partly lightened and no subject developed the adverse reactions of liver function damage and creatine phosphokinase elevation; the growth in terms of height and weight of the children was unaffected as well. This lipid-lowering therapy not only is suitable for adult patients with hyperlipidemia [31], but also is safe for this preschool-age (<6 years) compound heterozygous child.

Diagnosis of FH in China largely depends on clinical features, but patients with heterozygous mutations, such as the parents of the proband, often do not exhibit detectable clinical features. FH is detected in such patients only by measuring their cholesterol levels and by genetic diagnosis. In such patients, preventive treatment followed by early diagnosis is of great importance to avoid damage stemming from long-term elevation of cholesterol levels.

In summary, we successfully diagnosed FH genetically using next-generation sequencing methods and proved that it is a rapid, high-throughput, and efficient screening strategy. Briefly, TES of the 167 genes was sufficient and clinically useful for revealing genetic defects in patients with genetic dyslipidemia disease comprehensively.

## Supporting Information

Table S1 List of the genes captured in the present study.
(XLS)

Table S2 Data summary of the targeted exome sequencing.
(DOC)
