# Supplementary material for: Use of Targeted Exome Sequencing in Genetic Diagnosis of Chinese Familial Hypercholesterolemia
Source: PLoS One. 2014 Apr 10;9(4):e94697. doi: 10.1371/journal.pone.0094697 (PMC3983231; doi:10.1371/journal.pone.0094697)
Supplement: Table S2 — Data summary of the targeted exome sequencing. (DOC) [file pone.0094697.s002.doc]

**Table S2** Data summary of the targeted exome sequencing

| Sample | Proband |
| --- | --- |
| Raw_data(Mb) | 653.37 |
| Clean_data(Mb) | 649.73 |
| Aligned | 99.75 |
| Initial bases on target | 521023 |
| Base covered on target | 504935 |
| Coverage of target region | 96.90% |
| Total effective yield(Mb) | 519.06 |
| Effective sequence on target(Mb) | 333.62 |
| Fraction of effective bases on target | 64.30% |
| Average sequencing depth on target | 640.32 |
| Fraction of target covered with at least 4X | 94.70% |
| Fraction of target covered with at least 10X | 92.30% |
| Fraction of target covered with at least 20X | 89.30% |
| duplication rate(%) | 19.1131 |
